# Correlation between Constipation Symptoms and Abdominal CT Imaging: A Cross-Sectional Pilot Study

**DOI:** 10.3390/jcm12010341

**Published:** 2023-01-01

**Authors:** Mayuko Haraikawa, Tsutomu Takeda, Shotaro Oki, Mariko Hojo, Daisuke Asaoka, Tomoyo Iwano, Ryouta Uchida, Hisanori Utsunomiya, Nobuyuki Susuki, Daiki Abe, Atsushi Ikeda, Yoichi Akazawa, Kumiko Ueda, Hiroya Ueyama, Tomoyoshi Shibuya, Shuko Nojiri, Hidekazu Nagasawa, Masaru Suzuki, Ryohei Kuwatsuru, Akihito Nagahara

**Affiliations:** 1 Department of Gastroenterology, Juntendo University School of Medicine, Tokyo 113-0034, Japan; 2 Department of Gastroenterology, Juntendo Tokyo Koto Geriatric Medical Center, Tokyo 136-0075, Japan; 3 Department of Medical Technology Innovation Center, Juntendo University School of Medicine, Tokyo 113-0034, Japan; 4 Department of Radiology, Juntendo Tokyo Koto Geriatric Medical Center, Tokyo 136-0075, Japan; 5 Department of Radiology, Juntendo University School of Medicine, Tokyo 113-0034, Japan

**Keywords:** constipation, abdominal CT, CSS, BSFS

## Abstract

Evaluation of chronic constipation is important, although it is often difficult to satisfactorily treat due to the complex interplay of factors. This study aimed to determine whether the volume of intraluminal contents and lateral diameter of the colon measured from computed tomography (CT) images were correlated with the symptoms of chronic constipation and stool consistency. Consecutive patients who underwent the Constipation Scoring System (CSS), Bristol Stool Form Scale (BSFS) questionnaires and simple abdominal CT were selected retrospectively. The intestinal tract diameter at each site was measured, and the amounts of stool and gas in the intestinal tract were evaluated at five levels. Of the 149 study participants, 54 were males and 95 were females and their mean age was 72.1 years. In the right hemi-colon, CSS5 (Time) correlated significantly with gas volume (*p* < 0.01). In the left hemi-colon, stool volume correlated significantly with CSS2 (Difficulty), CSS3 (Completeness), CSS5 (Time) and CSS total (*p* < 0.05). The BSFS negatively correlated with gas volume and diameter in the right hemi-colon and with gas volume in the rectum (*p* < 0.05). CT findings including stool volume, gas volume and diameter correlated with some constipation symptoms and stool consistency. These findings may be useful in evaluating and treating constipation.

## 1. Introduction

The current super-aging society has become a problem worldwide. Chronic constipation is common, increasing with age and occurring in 2–28% of the population [1]. In general, due to constipation, quality of life (QOL) is impaired [2,3]. By significantly lowering QOL and impinging on an active lifestyle, constipation has been associated with systemic diseases including cardiovascular events, and appropriate treatment is necessary [4,5].

In evaluating constipation, assessment should be made of specific symptoms that are the most distressing and the medications being used [1]. Causes of constipation are difficult to determine in daily clinical practice. Rectal manometry, rectal balloon test [6], the colon transit time test, and defecography have been proposed for determining the cause of constipation [7]. Magnetic resonance (MR) defecography and dynamic MR pelvic imaging can be helpful for evaluation of the pelvic and rectal anatomy and motion, although they are difficult to perform and are available in few facilities.

Recently, it was reported that the length and volume of the adult colon could be measured by abdominal computed tomography (CT) and magnetic resonance imaging (MRI) [8,9,10]. The colon can accommodate varying amounts of intraluminal matter [11]. Reports have shown increased colon volume in those with functional constipation (FC) and irritable bowel syndrome (IBS) compared to healthy adults [12,13]. The volume of the colon in healthy individuals was reduced by about one third after defecation [14]. This indicates that colonic volume could be significantly increased by delayed defecation [15,16]. Thus, constipation has been evaluated with imaging, but the relationship between the results and abdominal symptoms with regard to bowel volume and localization of contents has not been examined. Therefore, we aimed to assess intestinal contents (such as stool and gas) that would dilate the intestines and determine differences in symptoms according to those contents. This is the first report showing whether the volume of intraluminal contents and the lateral diameter of the colon measured from CT images are correlated with the symptoms of chronic constipation and consistency of stool.

## 2. Materials and Methods

### 2.1. Patients

This was a retrospective clinical study performed in a single center to investigate the correlation between CT images and symptoms of constipation. Consecutive patients who were selected had undergone evaluation by the Constipation Scoring System (CSS) [17], an interview using the Bristol Stool Form Scale (BSFS) [18] and a simple abdominal and pelvic CT scan within 3 months before or after the interview date at the outpatient department of the Department of Gastroenterology, Juntendo Tokyo Koto Geriatric Medical Center, from January 2018 to October 2019.

### 2.2. Inclusion Criteria

Patients were included if all of the following information was available from their medical records: sex, age and body mass index (BMI); treatment with a proton pump inhibitor (PPI)/potassium-competitive acid blocker (PCAB); use of drugs to improve gastrointestinal functions (laxatives); results of the CSS and BSFS; and findings of simple CT of the abdomen.

### 2.3. Exclusion Criteria

Patients were excluded if they had a history of acute cerebrovascular, gastrointestinal, renal, coronary, hepatic or respiratory events, uncontrolled diabetes mellitus and a history of gastrointestinal surgery, inflammatory bowel disease, advanced gastrointestinal cancer, malignant lymphoma, leukemia, multiple myeloma or mental illness.

### 2.4. Assessments of Clinical Characteristics

The BMI was calculated by dividing body weight by body height in m^2^ (kg/m^2^). A PPI/PCAB user was defined as an individual who used any of the five types of PPI/PCAB (rabeprazole, lansoprazole, omeprazole, esomeprazole or vonoprazan) daily for more than 8 weeks. Laxative users were those who had taken any laxatives (bulk-forming laxatives, osmotic laxatives, carbohydrate laxatives, irritant cathartic, lubiprostones, linaclotides, elobixibat hydrate, herbal medicine) daily for more than 8 weeks.

### 2.5. Scoring of Abdominal Symptoms and Fecal Properties

We used the BSFS [18] to assess the condition of the participants’ feces. This diagnostic tool classifies stool density into seven categories as follows: 1. separate hard lumps, severe constipation; 2. lumpy and sausage-like, mild constipation; 3. sausage shape with surface cracks, normal; 4. like a smooth, soft sausage or snake, normal; 5. soft blobs with clear-cut edges, lacking fiber; 6. mushy consistency with ragged edges, mild diarrhea; and 7. liquid consistency with no solid piece, severe diarrhea.

The severity of constipation had been assessed according to the CSS [17]. The questionnaire contained various sections, including (1) Frequency of bowel movements, (2) Difficulty: painful evacuation effort, (3) Completeness: feeling incomplete evacuation, (4) Pain: abdominal pain, (5) Time: minutes in lavatory per attempt, (6) Assistance: type of assistance (laxatives, enemas or manual maneuvers), (7) Failure: unsuccessful attempts for evacuation per 24 h and (8) History: duration of constipation (years) with scores ranging from 0 (normal) to 4 (severe) for each section, with or without defecation assistance (range of 0 to 2). Individual scores are added together to get a global score. According to the global score, constipation can be categorized as mild (scores 1–5), moderate (6–10), severe (11–15) or very severe (15–30).

### 2.6. Image Evaluation and Analysis

We evaluated the results of simple abdominal and pelvic CT scans (axial image) performed within 3 months before or after the outpatient visits. There were no restrictions on oral intake prior to the CT scan.

Four board-certified gastroenterologists (MH1, TT, MH2, AN) evaluated the CT images. Delineative imaging quality was assessed for the following eight segments: cecum (ce), ascending colon (a), hepatic flexure (hf), transverse colon (t), splenic flexure (sf), descending colon (d), sigmoid colon (s) and rectum (re). The reviewers independently evaluated images using the window width. Window level settings were left fixed, and magnification and rotation were adjusted according to personal preferences. If the individual judgments did not match, judgment was made by consensus.

**Figure 1:**
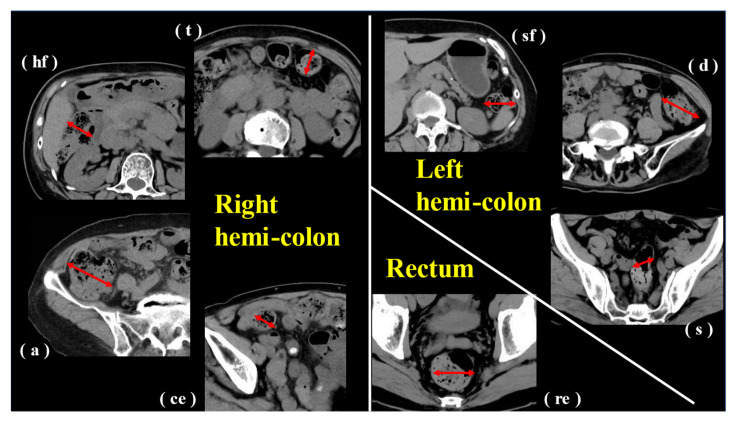
Simple CT image (axial image) of the abdomen and pelvis at each site of right hemi-colon, left hemi-colon and rectum. Diameter was measured at the most dilated site of the intestine (red line). (**ce**), cecum; (**a**), ascending colon; (**hf**), hepatic flexure; (**t**), transverse colon; (**sf**), splenic flexure; (**d**), descending colon; (**s**), sigmoid colon; (**re**), rectum.

**Figure 2:**
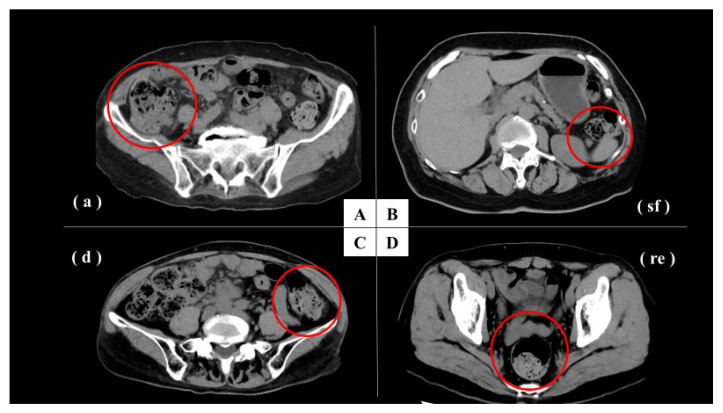
Representative cases. **A**: CT image was used to rate the stool volume as 4 and the gas volume as 2 (red circle). (**a**), Ascending colon. **B**: CT image was used to rate the stool volume as 1 and the gas volume as 2 (red circle). (**sf**), Splenic flexure. **C**: CT image was used to rate the stool volume as 3 and the gas volume as 2 (red circle). (**d**), Descending colon. **D**: CT image was used to rate the stool volume as 3 and the gas volume as 3 (red circle). (**re**), Rectum.

The most dilated sites of each intestinal segment (c, a, hf, t, sf, d, s, re) were selected and measured in all patients. For each of the identified maximum diameters in the intestinal tract, the intestinal contents (stool volume and gas volume) were evaluated (Figure 1). Content was rated on a 5-point scale from 1 to 5 (1, none or almost none; 2, less; 3, normal; 4, slightly more; and 5, more). We divided the segments into three groups by the area of blood flow control: right hemi-colon (c + a + hf + t), left hemi-colon (sf + d + s) and rectum. The average of c, a, hf and t was used for the right hemi-colon, and the average of sf, d and s was used for the left hemi-colon (Figure 2).

### 2.7. Statistical Analysis

We calculated the median value of BSFS and CSS. Statistical analysis was performed on scores of the BSFS and CSS and on averages of measured CT values and scores for the visual evaluation of CTs. The correlation between the CT image evaluation and constipation symptoms and various clinical parameters (gender, age, BMI, PPI/PCAB use, laxative use) was examined using Spearman’s correlation coefficient. For statistical analysis, statistical software SPSS for Windows, version 28.0 (SPSS, Inc., Chicago, IL, USA), was used. Correlation coefficient Rho < −0.20 or Rho > 0.20 with *p*-values of ≤0.05 indicated a statistically significant difference.

### 2.8. Ethical Considerations

The Juntendo Tokyo Koto Geriatric Medical Center Ethics Committee (No. 108-11; 18 December 2020) reviewed and approved this study. The ethical standards of the 2013 Declaration of Helsinki were followed.

## 3. Results

### 3.1. Study Participants’ Clinical Characteristics and Symptom Questionnaires

Participants’ mean age and mean BMI were 72.1 years (range: 38–89 years) and 22.6 kg/m^2^, respectively. Of the 149 participants, 54 were male and 95 were female. Of these, 40 were PPI/ PCAB users and 20 were laxative users. Median scores of the CSS and BFSF were 2.0 and 4.0, respectively (Table 1).

### 3.2. Image Evaluation and Analysis

Table 2 shows results of CT imaging and analysis as evaluated and scored by the reviewers. Stool volume on a five-point scale in the right hemi-colon was 3.2 ± 0.6, in the left hemi-colon was 2.4 ± 1.2 and in the rectum was 2.1 ± 0.9. Gas volume on a five-point scale was 3.1 ± 0.7 in the right hemi-colon, 2.3 ± 0.6 in the left hemi-colon and 2.5 ± 1.2 in the rectum. Average diameters (mm) of the intestinal tract were as follows: right hemi-colon, 29.3 ± 5.6; left hemi-colon, 19.3 ± 5.3 and rectum, 27.1 ± 11.0.

### 3.3. CT Image Evaluation and Correlation with Constipation Symptoms

**Figure 3:**
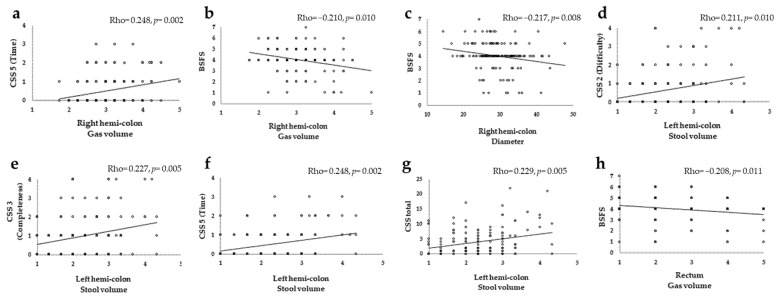
Scatter plot and the Spearman’s correlation coefficients of CT images and constipation symptoms. (**a**): Right hemi-colon gas volume and CSS5 (Time). (**b**): Right hemi-colon gas volume and BSFS. (**c**): Right hemi-colon diameter and BSFS. (**d**): Left hemi-colon stool volume and CSS2 (Difficulty). (**e**): Left hemi-colon stool volume and CSS3 (Completeness). (**f**): Left hemi-colon stool volume and CSS5 (Time). (**g**): Left hemi-colon stool volume and CSS total. (**h**): Rectum gas volume and BSFS. Correlation coefficient Rho (*p*-value): Rho < −0.20 or Rho > 0.20 with *p* < 0.05 indicates a significant difference. CSS, Constipation Scoring System; BSFS, Bristol Stool Form Scale.

Table 3 and Figure 3 show the Spearman’s correlation coefficients.

In the right hemi-colon, CSS5 (Time) correlated with gas volume (Rho = 0.248) and BSFS negatively correlated with gas volume (Rho = −0.210) and diameter (Rho = −0.217) (*p* < 0.05). BMI and gas volume (Rho = −0.273) were also negatively correlated.

In the left hemi-colon, stool volume correlated significantly (*p* < 0.05) with CSS2 (Difficulty) (Rho = 0.211), CSS3 (Completeness) (Rho = 0.227), CSS5 (Time) (Rho = 0.248) and CSS total (Rho = 0.229). BMI correlated negatively with stool volume (Rho = −0.264) and gas volume (Rho = −0.380).

In the rectum, gas volume correlated negatively with BSFS (Rho = −0.208) (*p* < 0.05) and BMI correlated negatively with stool volume (Rho = −0.203) and gas volume (Rho = −0.242).

No significant correlation was found for age, sex, PPI user or laxative user.

## 4. Discussion

Evaluation of CT findings showed that stool volume, gas volume and diameter were correlated with some constipation symptoms and stool consistency. The high gas volume in the right hemi-colon required more time for defecation as did the large volume of feces in the left hemi-colon. The latter was correlated with painful evacuation and the feeling of incomplete evacuation, which correlated with the total CSS. Higher gas volume in the right hemi-colon and rectum was correlated with lower BSFS, that is, it correlated with hard stools. No previous study that we have identified examined the correlation between results of the evaluation of each segment of the large intestine using CT and fecal characteristics with constipation symptoms.

According to Rome III, stool generally accumulates in the transverse colon where it absorbs water and electrolytes. If stools stagnate in the transverse colon, progressive peristalsis of the ascending colon provides feedback, and these stagnating stools are subsequently stored in the sigmoid colon by peristalsis. In other words, stool tends to accumulate in the transverse colon and sigmoid colon. A previous study of colonic movements in the cat showed that the right hemi-colon stores feces by retrograde movement of the contents from the anal to the oral side to promote water absorption. It was then shown that the right stool was slowly delivered to the descending colon and the stored stool was drained from the left hemi-colon [19]. A healthy descending colon has a smaller intestinal volume than those of the ascending and transverse colons [10]. An MRI study comparing pediatric patients with functional constipation with healthy control participants found that patients had higher bowel volume in the sigmoid colon and rectum than controls [16]. The use of colonic transit scintigraphy suggested that the transverse colon is the primary site for fecal storage [20].

No previous reports have used CT to visually evaluate the relationship between intestinal diameter, intestinal contents and intestinal gas volume to abdominal symptoms and stool characteristics. The present results showed a correlation between the location of stool and gas accumulation in the intestinal tract and abdominal symptoms. Under normal conditions, when there is no stool sensation, the rectum is void of feces. Since feces are mainly stored in the sigmoid colon and the left hemi-colon is the organ physiologically responsible for fecal storage, increased internal pressure in the left hemi-colon is one possible cause of symptoms such as the feeling of incomplete defecation and dyschezia.

In recent years, the relationships between rectal gas volume and rectal evacuation disorders have been studied [8]. Generally, the sigmoid colon and rectum have the function of storing feces, and stretching of the rectum stimulates the cerebral cortex via the sacral nerves, causing a bowel movement. Increased rectal gas is associated with hard stools and could cause impaired rectal evacuation. However, our study found no correlation between rectal diameter and abdominal symptoms. This may be due to hypoesthesia of the rectal mucosa [21].

In the current study, the amount and localization of intestinal tract contents were associated with abdominal symptoms as shown by abdominal CT. When stool was stored in the left hemi-colon, the patient experienced difficulty in defecating, sensation of residual stool, and required more time to defecate. It has been reported that the dilatation of the descending colon causes bloating and abdominal distention [22]. MRI evaluation of the colon for chronic constipation showed a correlation between the sensation of residual stool and the diameter of the descending colon [9]. In this study, the stool volume in the left hemi-colon was also correlated with the total CSS. Therefore, it is important to focus on CT findings of the left hemi-colon in patients with constipation and provide treatment according to symptoms including painful evacuation effort and the feeling of incomplete evacuation.

The BSFS correlated with stool transit time. A longer transit time in the intestinal tract was reported to result in harder stools and dyschezia [23]. In the present study, the amount of gas in the right hemi-colon correlated with the time required for defecation and was negatively correlated with the BSFS. An increase in the amount of gas in the right hemi-colon suggests the possibility of prolonged stagnation time of feces in the intestinal tract, resulting in hard stools. Findings by the BSFS were negatively associated with gas volume in the rectum. Therefore, when CT shows that the diameter of the rectum is dilated by intestinal contents, it is possible that the patient has hard stools. Ohkubo et al. reported the association of a low BSFS with a lower QOL, with BSFS type 4 being associated with the best QOL [24]. Thus, it is important for constipation treatment to be directed toward BSFS4.

BMI was negatively correlated with stool and gas volume. A higher BMI has been associated with increased diarrhea [25]. Manabe et al. reported that a high BMI accelerates colonic transit [26]. Low BMI may slow the colonic transit time, suggesting increased stool volume and gas volume. We have previously examined the association of sarcopenia and frailty with constipation and reported that muscle weakness was associated with higher CSS scores and lower nutritional status, and frailty was associated with higher CSS scores [27,28]. Although muscle strength was not examined in this study, elderly patients with a low BMI may be frail or have sarcopenia. Decreased function of the pelvic floor muscle group is one of the causes of functional difficulty in defecating [29]. Furthermore, prevention of generalized muscle weakness, including improvement of abdominal and pelvic floor muscles, is important, since weakness in abdominal muscles suppresses the increase in intra-abdominal pressure associated with a bowel movement. Further studies are needed to include an assessment of muscle strength and sarcopenia.

Among the study limitations were, first, its retrospective design, the small number of patients and its performance in a single center. Second, stool and gas volumes were assessed by a subjective visual evaluation, which is influenced by the experience of the diagnosing physician. Third, the evaluation of the intestinal tract was likely influenced by peristalsis, and final defecation and changes over time were not examined. In addition, the symptom questionnaires were administered on days separate from the day of the CT scan. Future studies should be performed on a larger number of cases and different constipation conditions should be analyzed.

In the future, abdominal CT can be expected to become an objective method to evaluate symptoms of constipation and formulate treatment strategies. Evaluation of constipation by CT may be useful not only in ruling out organic diseases but also in diagnosing gastrointestinal tract dysfunction.

## 5. Conclusions

In conclusion, there was a correlation between CT findings of stool and gas volume and constipation symptoms, as well as fecal characteristics in each region of the large intestine.

## Figures and Tables

**Table 1:** Clinical characteristics of study patients (n = 149).

Characteristics	Value
Sex (male:female)	54:95
Age (y), mean ± SD (range)	72.1 ± 10.4 (38–89)
BMI	22.6 ± 3.5
PPI/PCAB	Non-users: 109, Users: 40
Laxative	Non-users: 129, Users: 20
Questionnaires, median (IQR)	
CSS	2.0 (1–6)
BSFS	4.0 (4–5)

SD, standard deviation; BMI, body mass index (kg/m^2^); PPI, proton pump inhibitor; PCAB, potassium-competitive acid blocker; IQR, interquartile range; CSS, Constipation Scoring System; BSFS, Bristol Stool Form Scale.

**Table 2:** Evaluation of CT images and analysis.

Anatomic Location	CT Findings		
	Stool Volume	Gas Volume	Diameter (mm)
Right hemi-colon	3.2 ± 0.6	3.1 ± 0.7	29.3 ± 5.6
Cecum	3.6 ± 0.8	3.2 ± 0.7	35.8 ± 9.3
Ascending colon	3.3 ± 0.8	3.1 ± 0.7	32.6 ± 8.1
Hepatic flexure	3.1 ± 0.8	3.0 ± 0.8	26.8 ± 7.4
Transverse colon	2.8 ± 0.9	3.0 ± 1.8	22.7 ± 6.6
Left hemi-colon	2.4 ± 1.2	2.3 ± 0.6	19.3 ± 5.3
Splenic flexure	2.5 ± 0.9	2.7 ± 0.9	22.2 ± 7.0
Descending colon	2.3 ± 2.1	2.1 ± 0.8	18.2 ± 6.6
Sigmoid colon	2.3 ± 1.0	2.2 ± 0.9	17.4 ± 6.1
Rectum	2.1 ± 0.9	2.5 ± 1.2	27.1 ± 11.0

Average ± standard deviation.

**Table 3:** Correlation between results of evaluation of CT images and constipation symptoms. Rho (*p*-value).

		CSS1 (Frequency)	CSS2 (Difficulty)	CSS3 (Completeness)	CSS4 (Pain)	CSS5 (Time)	CSS6 (Assistance)	CSS7 (Failure)	CSS8 (History)	CSS Total	BSFS
Right hemi-colon	Stool volume	0.041 (0.618)	0.150 (0.067)	0.011 (0.893)	−0.027 (0.746)	0.150 (0.068)	−0.099 (0.229)	0.034 (0.678)	0.039 (0.636)	0.047 (0.567)	−0.138 (0.094)
	Gas volume	0.120 (0.145)	0.142 (0.084)	0.073 (0.376)	0.098 (0.235)	0.248 * (0.002)	−0.116 (0.160)	0.073 (0.381)	0.055 (0.507)	0.122 (0.139)	−0.210 * (0.010)
	Diameter	−0.074 (0.368)	0.012 (0.885)	0.016 (0.844)	−0.027 (0.742)	0.062 (0.449)	−0.112 (0.175)	0.019 (0.818)	−0.066 (0.423)	−0.056 (0.497)	−0.217 * (0.008)
Left hemi-colon	Stool volume	0.142 (0.008)	0.211 * (0.010)	0.227 * (0.005)	−0.012 (0.885)	0.248 * (0.002)	0.058 (0.483)	0.036 (0.483)	0.154 (0.060)	0.229 * (0.005)	−0.106 (0.198)
	Gas volume	0.169 (0.039)	0.146 (0.075)	0.102 (0.217)	−0.009 (0.914)	0.101 (0.219)	0.081 (0.326)	0.057 (0.491)	0.043 (0.598)	0.149 (0.070)	0.050 (0.543)
	Diameter	0.078 (0.347)	0.169 (0.039)	0.172 (0.036)	0.061 (0.457)	0.078 (0.344)	0.036 (0.660)	0.112 (0.176)	0.089 (0.278)	0.189 (0.021)	−0.099 (0.231)
Rectum	Stool volume	0.100 (0.226)	0.025 (0.762)	0.047 (0.572)	−0.052 (0.531)	0.006 (0.941)	−0.170 (0.038)	−0.105 (0.205)	0.055 (0.503)	0.078 (0.344)	−0.116 (0.159)
	Gas volume	0.032 (0.701)	0.042 (0.614)	0.007 (0.933)	−0.119 (0.148)	0.062 (0.456)	−0.158 (0.055)	−0.033 (0.693)	−0.032 (0.696)	−0.035 (0.672)	−0.208 * (0.011)
	Diameter	0.019 (0.822)	0.066 (0.421)	−0.078 (0.344)	−0.113 (0.168)	0.054 (0.515)	−0.100 (0.225)	−0.025 (0.760)	0.011 (0.893)	0.055 (0.502)	−0.139 (0.090)

Correlation coefficient Rho (*p*-value): Rho < −0.20 or Rho > 0.20 with * *p* < 0.05 indicates a significant difference. CSS, Constipation Scoring System; BSFS, Bristol Stool Form Scale.

## Data Availability

Not applicable.
